# Crossing the Valley of Death in Spinal Cord Injury: Learning from Successful Translators

**DOI:** 10.1089/neur.2025.0029

**Published:** 2025-04-14

**Authors:** Vieri Failli, Stephen M. Strittmatter, Martin E. Schwab, Aileen J. Anderson, Angela Ruban, Hassan Al-Ali, Armin Curt, Adrien Cohen, Jane T.C. Hsieh

**Affiliations:** ^1^ Wings for Life, Salzburg, Austria.; ^2^ Cellular Neuroscience, Neurodegeneration and Repair Program, Departments of Neurology and of Neuroscience, Yale University School of Medicine, New Haven, Connecticut, USA.; ^3^ Institute for Regenerative Medicine, University of Zurich, Schlieren, Switzerland.; ^4^ Sue and Bill Gross Stem Cell Research Center, University of California, Irvine, California, USA.; ^5^ Sackler Faculty of Medicine, Medicine and Health Sciences, Sagol School of Neuroscience, Tel Aviv University, Tel-Aviv, Israel.; ^6^ The Miami Project to Cure Paralysis, Department of Neurological Surgery, Sylvester Comprehensive Cancer Center, Peggy and Harold Katz Drug Discovery Center, Katz Family Division of Nephrology and Hypertension, Department of Medicine, and Frost Institute for Data Science and Computing, University of Miami Miller School of Medicine, Miami, Florida, USA.; ^7^ Spinal Cord Injury Center, Balgrist University Hospital, University of Zurich, Zurich, Switzerland.; ^8^ SCI Ventures, London, United Kingdom.

**Keywords:** collaborative funding, spinal cord injury, translation medicine, translational science, venture philanthropy

## Abstract

A straightforward path to successful scientific translation remains uncharted, particularly in a complex progressive condition such as spinal cord injury (SCI), which affects multiple body functions simultaneously. Evolving regulatory requirements add to the complexity and expense of attaining a treatment that is both safe and efficacious. Although rare, there are examples of SCI scientists who have successfully navigated the “valley of death” from discovery science to completed clinical trials. This article reflects the translational journey of five SCI scientists who have encountered similar and different scenarios while striving to launch or complete a clinical trial. Learning from these experiences has identified lessons learned and gaps, particularly with respect to funding and support for SCI translation.

**Figure:**
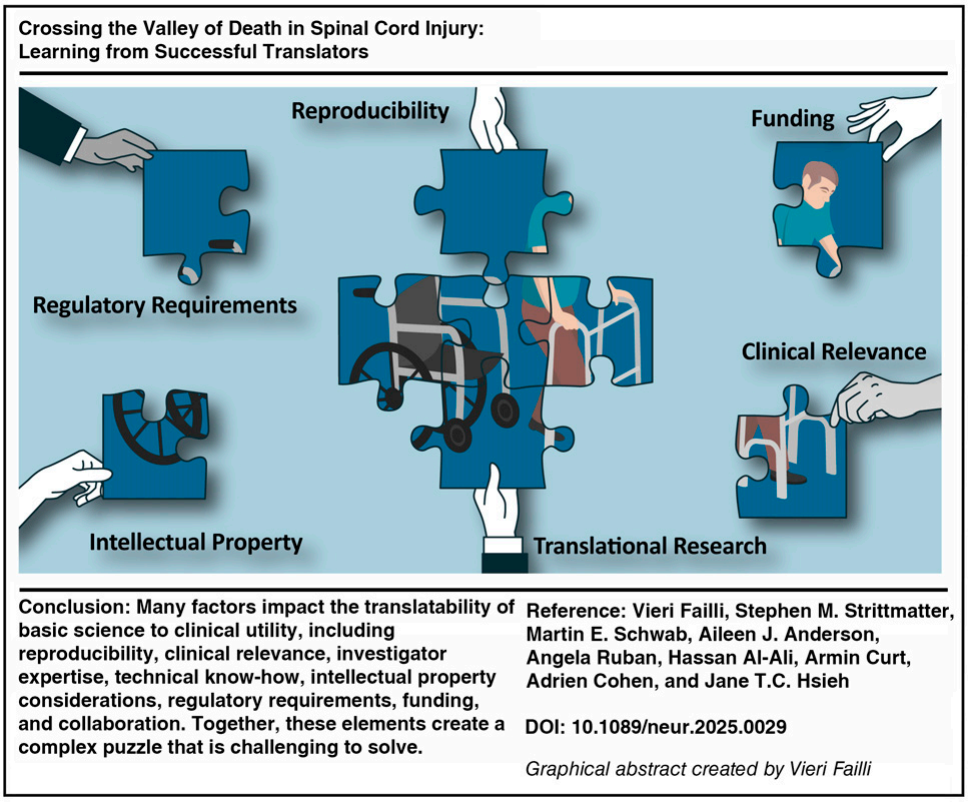


## Introduction 

Spinal cord injury (SCI) is a complex, multifaceted, and progressive condition that can lead to lasting sensory and motor deficits as well as various chronic complications. Studies in animal models over the last 30+ years have yielded important insights into the neurobiological mechanisms of lesion-induced degeneration, rehabilitation-dependent functional recovery and experimental approaches to enhance axonal regeneration and compensatory plasticity. Sadly, despite these extensive research efforts that have led to significant therapeutic advances in animal models, human SCI remains incurable. The transition from discovery science to proven clinical treatments is not only long, but also hindered by a significant gap between bench research and clinical validation. This gap is often called the “valley of death,” where many discoveries are unable to overcome the numerous hurdles required to demonstrate safety and effectiveness for human use.

Discovery research involves high-risk investment for the future where utility may not be realized for decades. However, preclinical research needs to be purposeful if successful translation to clinical utility is to be achieved efficiently. Purposeful preclinical research does not always follow the linear translational process outlined in the peer-reviewed literature.^1^ Unpredictable feedback loops between various disciplines are to be expected and the predictive value of animal models has been debated for at least two decades.^2,3^ Additionally, multiple other factors impact the translatability of basic science to clinical utility, such as reproducibility, clinical relevance, investigator base, technical expertise, intellectual property, regulatory requirements, funding, and collaboration, creating a difficult-to-solve puzzle.^4^ The high cost of complex translation affects each step of the process and coordination, partnerships, and targeted innovation in funding are necessary to further reduce the timeline for effective therapeutic development.

With so many hurdles to traverse in the valley of death, understanding successful real-life translational journeys may elucidate critical factors helpful to those in the process of translating or planning to translate their work. The Wings for Life is a spinal cord research foundation founded in 2004 that supports preclinical and clinical research. In light of numerous requests from scientists and clinicians working in this field who were unable to find a composite of information on clinical translation in SCI neurorestoration, a one-day workshop was organized during the 2024 scientific meeting, gathering several scientists who presented their translational journeys. The limited perspectives presented reflect the relatively few that have successfully translated preclinical findings into completed neurorestorative clinical trials in SCI. This article summarizes the main themes and suggestions for those willing to venture into this journey, acknowledging that experiences shared are not necessarily recommendations. The workshop was also streamed live, and hundreds of participating scientists across two continents. Here, we present the translational journeys of three investigators whose scientific work has been implemented in completed clinical trials. Additionally, two investigators present their work in progress as they prepare for clinical trials. Finally, two funding mechanisms are described to expedite translation toward therapeutic development in SCI.

## Part 1: Successful Translational Journeys from Discovery Science to Completed Clinical Trials

### Session 1

**Background:** Dr. Stephen Strittmatter began his research career by studying the molecular basis of axonal guidance while interning in adult neurology at Massachusetts General Hospital. After three decades of bench and preclinical science funded by various philanthropic sources, as well as institutional and foundation grants, a Nogo Receptor decoy (AXER-204) was prepared to be tested in a clinical trial. This scientific work performed at Yale University was licensed to ReNetX Bio, which sponsored the clinical trial through a collaborative funding model with the Wings for Life Accelerated Translational Program and the National Institute of Neurological Disorders and Stroke (NINDS). The RESET (Renetx Safety Efficacy and Tolerability) trial of AXER-204 (Soluble Nogo-Receptor-Fc decoy) treatment was intended to promote neural repair in chronic SCI.^5^

**Summary:** The process of drug development for SCI begins with identifying a suitable molecular target. Once identified, various therapeutic agents such as antibodies, peptides, and decoy receptors are developed, focusing on ligand-binding domains. An essential early step is the creation of a target product profile (TPP), which, for chronic cervical SCI, includes considerations like repeated lumbar puncture (LP) delivery (excluding pump methods) and the enhancement of motor recovery. It’s crucial to understand that the molecule’s development is influenced by the intended route and mode of delivery. Preclinical studies are significant, and in the case of the Nogo protein and its receptor, an extensive range of studies, including a meta-analysis of modulation effects, was conducted. Although nonhuman primate studies are useful, in this instance, they were conducted postclinical protocol development. For AXER-204, the translation to clinical application marked the first step, which is notably costly and time-consuming, relying on various funding sources such as venture capital (VC), investors, NIH (National Institutes of Health) funds, and National Center for Advancing Translational Sciences (NCATS). The creation of a good manufacturing practice (GMP) master cell bank is an extended process, and the production of a large quantity of drug substance and drug product is complex and beyond the capacity of all, or nearly all, academic settings. Preclinical toxicology, including dose-finding and acute and chronic good laboratory practice (GLP) studies, is vital but cannot be pursued solely in academic contexts. Systemic exposure following intrathecal (IT) delivery and the maximum duration of exposure in nonhuman primates were evaluated using various animal models, with no adverse events reported. Selecting the correct dose for clinical trials is a delicate balance, often narrowed down to a single dose for practicality. Preclinical documentation of dose-dependence is critical, but translating doses across species is challenging, even when cerebrospinal fluid drug level monitoring is included. Pharmacokinetics (PK) and pharmacodynamics (PD) are complicated to ascertain and are subject to stringent FDA (Food and Drug Administration) requirements. The emphasis in the regulatory process lies heavily on safety rather than efficacy. The FDA demanded extensive toxicology data, including the effects of the drug when delivered peripherally, despite IT administration being the primary route. The manufacturing details of the protein were scrutinized, with a particular focus on stability and aggregation. Institutional review boards (IRBs), particularly central IRBs, play a role in streamlining the process for multi-center trials, a practice encouraged by the NIH. The FDA’s response times can be slow, typically taking 60 to 90 days, with minimal guidance provided. Fast-track designation may facilitate more interactive and prompt communication with regulatory bodies. For spinal cord injuries, the FDA has not established clear registrational endpoints or defined clinically meaningful outcomes, making drug approval for SCI recovery particularly ambiguous. Clinical trial design must be considered years in advance, shaping preclinical studies and toxicology programs. Clinically meaningful endpoints for SCI are a “moving target,” requiring careful consideration. The statistical analysis plan must be locked in ahead of time and informed by reliable natural history data to accurately power a clinical trial, although currently, there is a lack of validated options.

**Key points:** Commercialization typically begins after the early academic stages, as later phases like dose-response, PK/PD studies, and GMP/GLP production require a commercial approach, possibly with grant support. Protecting intellectual property during the academic stages is crucial to ensure a smooth transition to commercialization.

### Session 2

**Background:** While studying the cellular and molecular mechanisms of nerve fiber growth during the regeneration of injured nerve fibers in the spinal cord and brain, Dr. Martin Schwab identified a novel CNS (central nervous system) protein, Nogo-A, that inhibited nerve fiber outgrowth. This discovery led to the development of function-blocking antibodies against Nogo-A. Several of these antibodies enhanced anatomical nerve fiber growth and regeneration as well as functional recovery in rodent and nonhuman primate models of SCI. A human anti-Nogo-A antibody was applied in two clinical trials. The most recent, a multicenter European Phase 2 clinical trial, was completed in July 2023.^6^

**Summary:** As in most cases, the story began with academic bench research, where this novel concept was discovered and its mechanism of action investigated via preclinical proof of concept in animal models. The project then moved to preclinical drug development, which involved creating a drug suitable for human use, significantly different from the reagents used in an academic laboratory. Drug development is a complex field, involving chemistry, manufacturing, and control (CMC), which is quite foreign to most academic researchers. The project required the creation of fully human anti-Nogo antibodies, a step that involved overcoming significant technical challenges in stability, yield, and immunological properties, and was essential for patent protection. Patents are of utmost importance, as they are crucial for attracting investors. However, the process of writing patents requires specialized legal help, which is costly and time-consuming. Preclinical toxicology studies are also a significant step in the drug development process, often requiring studies in two species, including costly tests in nonhuman primates. There are numerous challenges faced in drug development, such as the expertise and procedures that are not available in a university setting, requiring external suppliers and consultants. Toxicology, formulation, and bioanalysis (quantitative measurement of a compound or its metabolite in biological fluids) are crucial aspects that require validation and rigorous testing, all part of the extensive documentation necessary for regulatory approval. As with the previous (RESET) trial, an Investigational Medicinal Product Dossier (IMPD), a very detailed file of documents of hundreds of pages, had to be generated to provide information to the regulatory agencies about the drug development process. The purpose of an IMPD is to ensure that the drug development process of the manufacturer is efficient, according to GMP and provides all the required relevant medical, safety, and scientific information for evaluating the potential drug. Other aspects had to be considered, such as medical need, potential patient population, market size, competitive landscape, and potential funding sources. The process culminated in a clinical trial application, a key regulatory step that includes a comprehensive package of documents, such as the TPP, the IMPD, the investigator’s brochure, and the detailed clinical trial protocol. The Nogo-A Inhibition in acute Spinal Cord Injury (NISCI) trial, which was a Phase 2 clinical trial, involved 14 SCI centers across five European countries. It had specific inclusion and exclusion criteria and subdivided patients into subcohorts to allow for more accurate analysis of the results. The trial had one primary readout, the upper extremity motor score, and, in addition to safety, several secondary and exploratory readouts, all of which required standardized operating procedures and extensive data handling. Dr. Schwab noted that there is freedom in planning as many exploratory readouts as one wishes, but with obvious impacts on data collection and its associated costs. After careful consideration all should be included in standard operating procedures (SOPs), which are fixed and trained in all the centers. In multicenter trials such as NISCI, SOPs ensure that all participating sites follow the same procedures, minimizing variations in data collection and ensuring reliable results. Generally, a multicenter Phase 1 trial costs €5 − 7 million, while a Phase 2 trial can cost ten to 25 million or more. The NISCI Phase 2 trial was run as an investigator-initiated, academically supported trial. NISCI was financed by a program of the European Union, the Wings for Life, and other sources, totaling roughly €10 million.

**Key points:** Dr. Schwab’s journey from neuroscience to drug development and clinical trials illustrates the complex and costly process of bringing a therapeutic antibody from the academic bench to clinical trials, highlighting the multidisciplinary efforts, regulatory challenges, and financial demands of such an endeavor.

### Session 3

**Background:** Dr. Aileen Anderson’s academic career includes a focus on human CNS stem cell engraftment in the injured CNS and mechanisms for the promotion of neurological recovery. A culmination of two decades of research led to the use of human central nervous system stem cells (hCNS-SC), which have been shown to improve functional recovery after being grafted into contused spinal cords of NOD-Scid mice, in two clinical trials.^7–9^

**Summary:** Compared to traditional pharmacotherapeutics, cell therapies have an additional level of complexity regarding potential risks. Since regulatory agencies are more concerned with risk than efficacy, their scrutiny of stem cells is far greater. This is especially true for embryonic stem cells, which can differentiate into any cell type and therefore have greater tumorigenic potential. Dr. Anderson’s work is based on tissue-derived human neural stem cells capable of generating neurons, oligodendrocytes, and astrocytes. It is important to note that these are distinct from the cells developed by the company StemCells, Inc. Despite differences in isolation methods, the resulting cells share common features, such as the ability to form neurospheres and self-renew. These cells have the potential for pleiotropic, multitarget activity, which can exert combined beneficial and detrimental effects. There is potential for donor as well as batch variations, which can impact the final product. This introduces additional complexity, considering that transplanted cells persist and interact with the host environment in diverse ways. The project began in 2002, culminating in a Swissmedic-approved thoracic trial in 2010 and subsequent preclinical studies aiming to extend applications to cervical and chronic injuries. Funding transitioned from NIH grants to larger translational grants and philanthropy, with significant support from the California Institute for Regenerative Medicine (CIRM), enabling a latitude in development not available elsewhere. There are numerous challenges and intricacies associated with scaling up cell production for clinical trials. These include the need to maintain consistent cell characteristics despite batch-to-batch variations, which can be a major cause of failure in cell therapy development. The FDA requires surrogate assays to demonstrate consistency across batches, which are tested *in vitro* and do not necessarily inform on the *in vivo* activity. Cell production scaling introduces unexpected problems as evidenced by failures such as the Osiris Therapeutics Phase 3 trial failure. It is also essential to understand that both *intrinsic* and *extrinsic* factors control stem cell behavior. Therefore, it is important to understand the “niche” environment into which the cells are transplanted, because it varies depending on whether it is intact, damaged, or under repair. To facilitate quality control, Dr. Anderson developed a predictive gene expression profile that can anticipate the efficacy of cell lines produced under GMP conditions. Process parameters such as seed bank passage and seeding density are also critical, as they can significantly impact the volume of the final product. It is necessary to establish, as early as possible, a TPP, which includes disease indication, expected biological activity, efficacy endpoints, safety profile, dosing regimen, and route of delivery. These parameters are then reviewed by regulatory agencies, which often respond by requesting more data. As expected, the costs associated with cell therapy production are enormous. For example, even in the academic center based GMP facilities used for this project, where costs are kept to a minimum, the current second manufacturing run still amounts to a staggering $750,000.

**Key points:** While the TPP should be developed as early as possible, Dr. Anderson suggests delaying the creation of a company until after an investigator-initiated trial (IIT) has demonstrated the therapy’s potential. This strategy aims to preserve the integrity and targeted direction of the therapy’s development, in contrast to the risk of companies pivoting away from the original therapeutic indication after acquiring intellectual property. However, this scenario is feasible only if funding is available, which in this case was possible via CIRM’s support.

## Part 2: In-Progress Translation Toward a Clinical Trial

### Session 4

**Background:** A.R. from Tel Aviv University, has devoted her academic career to therapeutic approaches intended to improve the environment of spinal cord lesions. These approaches aim to reduce axonal degeneration, glial scar formation, and neuronal death, as well as to increase the capacity for axonal regeneration. A new anti-glutamatergic drug administered to animal models with spinal cord injuries is at the final preclinical drug development stage. If successful, this investigational drug is being developed for administration by first responder as a unique emergency neuroprotective agent. Dr. Ruban anticipates initiating a Phase 1 clinical trial by 2025.

**Summary:** With her background as a clinical pharmacologist and educator in drug development, Dr. Ruban's approach was methodical, leveraging her knowledge to design a roadmap from inception to market. In the academic phase, Dr. Ruban’s lab conducted six years of extensive preclinical research, costing around $2 million. This research included *in vitro* screening, *in vivo* efficacy in various SCI models (hemisection, contusion, and compression), PK and PD analysis, dose-response analysis, therapeutic window determination, treatment protocol optimization, and preliminary toxicology. This groundwork facilitated the company’s founding when the preclinical study was complete. Dr. Ruban’s treatment, based on recombinant enzymes, aims to boost the body’s natural healing systems and prevent secondary damage after SCI. She demonstrated significant functional recovery in treated mice compared to untreated ones, highlighting the treatment’s neuroprotective effect. Dr. Ruban emphasized the importance of characterizing compounds early, including stability, solubility, mode of action, applied therapeutic window, and PK parameters. This characterization informs treatment protocols and satisfies investor inquiries, reducing uncertainty and investment risk. It is equally important to include several animal models to identify the target demographic for clinical trials. Dr. Ruban advised scientists who are uncertain of their project’s stage (Technology Readiness Level [TRL]) to apply for an Innovation Task Force Briefing (PreIND) meeting to understand the regulatory requirements (see Table 1). This is done via the European Medicines Agency and is free of charge. As an example, Dr. Ruban submitted via this program a summary of the preclinical results on efficacy, a detailed treatment protocol (doses, route of administration, and therapeutic window), a protocol for the toxicology studies, and a draft protocol for the final formulation. Funding for Dr. Ruban’s work initially came from small academic grants and then from larger entities such as the Israeli Innovation Authority, the Israel Department of Defense, and the Wings for Life, which supported the completion of the proof of concept. The US Department of Defense funded alternative formulations, helping to determine the optimal one for further development. NeuroHagana plans to develop the treatment in Europe, and Dr. Ruban underscored the importance of understanding IP (Intellectual Property) status, TTO (Technology Transfer Office) negotiations, and potential royalty obligations as these impact investor decisions. Additional support came from the European Innovation Council (EIC) Accelerator Program, which funds high-risk, high-impact innovations, detailing the application process and the potential for substantial financial backing. It provides blended finance to support development (TRL 4 to 8), deployment, and scale-up (TRL 9). Grant support for early-stage companies is capped at a maximum of €2.5 million, while companies that are at the clinical trial phase can receive investments up to €50 million, usually in the form of direct equity or quasi-equity. NeuroHagana applied for blended finance, first receiving a grant to reach stage TRL 5 within two years and already applying for the investment component to move to the clinical trial stage.

**Table 1.** Essential Elements of PreIND Meeting Request Letter

1. Product name and IND number	Name of the product and IND application number, if previously assigned.
2. Chemical details	Chemical name, description of the molecular entity, established name, and structure.
3. Regulatory pathway	Proposed regulatory pathway (e.g., FDA 505(b)(1) for novel drugs, 505(b)(2) for referencing existing studies).
4. Indications	Proposed therapeutic indication(s) for the product.
5. Meeting type	Type of meeting requested (e.g., preIND—type B).
6. Additional details	Other relevant information such as combination product details or pediatric study plans, if applicable.
7. Purpose statement	Statement of purpose, including completed and planned studies or data to be discussed during the preIND meeting.
8. Agenda	Proposed agenda with anticipated time requirements and designated speakers for each agenda item.
9. Questions	Proposed questions covering areas such as preclinical, clinical, and chemistry, manufacturing, and controls (CMC).
10. Attendees	List of company attendees, including consultants, with their titles and affiliations.
11. FDA participants	FDA staff requested for participation in the meeting.
12. Information package delivery date	Date when the preIND meeting information package will be delivered to the regulatory office.
13. Meeting schedule	Proposed date/time options and suggested format (in-person, videoconference, or written response only) for the meeting.

IND, FDA, Food and Drug Administration.

IND (Investigational New Drug Application), FDA (Food and Drug Administration).

**Key points:** Dr. Ruban’s strategy involved a clear, step-by-step roadmap for drug development from academia to market, informed by preclinical rigor, strategic planning, and proactive regulatory engagement. The company is currently pursuing funding through the EIC to advance to clinical trials, illustrating a successful transition from academic research to potential commercialization. Dr. Ruban suggested that there is no ideal moment to start the creation of a company since it mostly depends on personal preference as well as financial needs.

### Session 5

**Background:** Understanding the signaling pathways that regulate axon growth and identifying targets for drug-mediated axon regeneration are research areas that Dr. Hassan Al-Ali is undertaking at The Miami Project to cure paralysis. Dr. Al-Ali utilizes three main strategies to identify drug targets for promoting axon growth: phenotypic screening, target-based profiling, and machine learning. Using knowledge of multiple targets, Dr. Al-Ali’s team then identifies and develops molecules with multitarget activity (polypharmacology), which results in high efficacy. After 14 years of work, Dr. Al-Ali’s group is ready to initiate preclinical testing of their top candidate drug.

**Summary:** Dr. Al-Ali, an active researcher in the field of drug discovery, is familiar with the so-called “valley of death,” a metaphor for the resource gap in technology development that roughly aligns with the drug development stages from lead optimization to early clinical testing. The width of this valley fluctuates with the economic climate, typically widening during economic downturns when attention shifts towards later-stage assets. Dr. Al-Ali's journey began with a groundbreaking discovery: a molecule capable of coengaging multiple targets to regulate axon growth.^10,11^ This compound demonstrated significant effects on neurite outgrowth *in vitro* and axon growth *in vivo*. Poised to move from discovery to development, Dr. Al-Ali and his team secured funding through the Wallace H. Coulter Center at the University of Miami, designed to propel promising science toward translational research. With this support, they initiated chemistry work and preliminary PKs and toxicology studies via contract research organizations (CROs). Parallel to these efforts, the compound was shared with collaborators and tested extensively to shed light on delivery requirements, timing postinjury, and therapeutic windows, all yielding positive data that underpinned therapeutic potential. This was crucial since the multitarget nature of the molecule presented a unique chemistry challenge, in addition to the existing hesitation around SCI drug development. These promising results allowed Dr. Al-Ali’s team to successfully compete for funding from the Blueprint Neurotherapeutics Network (BNN), an NIH program offering nondilutive funding and a comprehensive support system mimicking a biotech environment, propelling assets from basic research into Phase 1 clinical trials. The BNN forms a lead development team around the principal investigator team, providing a biweekly consultative framework and access to a network of CROs. Dr. Al-Ali started this work as a postdoctoral fellow in the lab of Dr. John Bixby and Dr. Vance Lemmon; the three have continued to work together on this program and were later joined by Dr. Jae Lee. Reflecting on his 14-year journey, Dr. Al-Ali recounted critical collaborations and negotiations, such as securing freedom to operate with a compound owned by Roche. Each step was pivotal, from the initial idea of using polypharmacology in 2010, discovering targets and compounds in 2012–2013, to the first demonstration of structure-activity relationship and PKs/toxicology studies in 2019. As the team gears up for IND-enabling studies, Dr. Al-Ali offered advice for those embarking on a similar path. He emphasized the importance of developing a TPP early on to guide research and development decisions, thereby minimizing tangential efforts and costly missteps during early development. Understanding PK, PD, and target engagement is also critical for evaluating the therapeutic potential of a compound and is a prerequisite for serious consideration by co-development industry partners and funding programs such as the BNN. Dr. Al-Ali also highlighted the criticality of selecting the appropriate drug delivery route, which can have profound implications on a drug’s efficacy and bioavailability. Furthermore, he stressed the importance of securing intellectual property through patents, without which commercial translation is impossible, and underscored the need for strategic planning, including the consideration of whether to pursue entrepreneurship, continue as an academic scientist, or some hybrid of the two.

**Key points:** In summary, Dr. Al-Ali acknowledged the necessity of surrounding oneself with a capable team that complements one’s expertise, particularly in areas outside the typical scientific purview. He advocated for engaging with FDA consultants and business development experts early in the process to ensure efficient progression toward the goal of bringing a therapeutic to market.

## Part 3: Novel Funding Mechanisms for Therapeutic Development in Spinal Cord Injury

### Session 6

**Background:** The Wings for Life Research Foundation was founded in 2004 with the goal of finding a cure for SCI. After 12 years of funding mostly discovery and preclinical research, the Wings for Life recognized the need to assist scientists in translating their preclinical findings into clinical trials. As such, the Accelerated Translation Program (ATP) was formed and funded its first clinical trial in 2016. Dr. Armin Curt, Chief and Director of the SCI Center at Balgrist Hospital in Zurich, is the ATP’s Scientific and Clinical Director. ATP applications must be interdisciplinary, collaborative, innovative in clinical trial design, and include integrated discovery research. The ATP is unique as it offers a support network to help investigators in multiple aspects of translation; and does not have predefined timeline or budget restrictions. At present, no other private SCI Foundation regularly receives applications for funding of clinical translation in SCI neurorestoration.

**Summary:** The Wings for Life, an organization dedicated to advancing spinal cord research, has instituted an ATP to bridge the gap between preclinical studies and early clinical trials. The ATP, a unique SCI Foundation funding program, is designed to support the transition through Phase 1 and Phase 2 trials, focusing particularly on neural repair at the spinal cord level, with the goal of achieving clinically meaningful outcomes such as motor, sensory, and autonomic function recovery that can facilitate clinical improvement for patients. At the heart of ATP’s mission is to expedite the journey from laboratory research to tangible patient outcomes. Emphasizing the treatment and rehabilitation of spinal cord injuries, the program prioritizes clinical trials that demonstrate the potential for real-world impact, such as motor and sensory recovery, and the restoration of autonomic functions. ATP’s unique approach is characterized by its flexibility, notably its lack of application deadlines. This feature underscores the program’s commitment to supporting research endeavors in a time-sensitive manner, acknowledging the inherent challenges and delays often encountered in the trial process. By removing rigid timelines, ATP offers researchers the ability to progress at a pace conducive to their project’s needs. The foundation has established a support network as part of ATP to assist investigators in addressing challenges that arise during the research and trial phases. This network provides a platform for the exchange of ideas and strategies, fostering a proactive environment where potential issues can be anticipated and addressed early on. ATP encourages collaboration with start-up companies and IITs, fostering an ecosystem where emerging businesses and research initiatives can thrive. The program also endorses co-funding strategies, recognizing the importance of securing additional financial resources for the successful execution of clinical trials. An executive oversight committee is integral to the ATP, engaging in regular dialogue with research teams and companies. This committee’s role is to identify and navigate through the trials’ challenges, offer guidance, and ensure that the foundation remains well-informed and satisfied with the progress of supported projects. To engage with ATP, researchers must present a comprehensive plan, a solid team, and a clearly defined clinical goal. The foundation stresses the importance of achieving tangible outcomes that benefit patients directly, rather than merely proving scientific concepts. To this end, the Wings for Life seeks interdisciplinary approaches that inform clinical trial designs, including meticulous considerations of measurement methods, patient stratification, and clear objectives. Additionally, the ATP advocates for inclusive protocol designs that not only target primary endpoints but also facilitate further learning. This could involve the exploration of biomarkers, drug interactions, and the development of novel indicators that may be instrumental for subsequent trials. The ATP’s iterative dialogue process is outlined on the foundation’s website, providing guidance on application requirements. The program’s emphasis on interdisciplinarity ensures that trials are comprehensive and informed, considering the multifaceted nature of spinal cord injuries and the various factors that can influence patient outcomes.

**Key points:** In conclusion, the ATP program by the Wings for Life represents a dedicated effort to support early-stage clinical trials, providing a framework that is both supportive and adaptable. It has already facilitated several trials and continues to cultivate an environment conducive to educational exchange and innovation in the field of neural repair. The take-home message is clear: ATP is committed to fostering early clinical translation with a focus on interventions that have the potential to significantly improve the lives of patients with SCIs.

### Session 7

**Background:** SCI Ventures is the first venture philanthropy fund (VPF) created to catalyze new treatments for paralysis. The fund invests in early-stage companies, and recycles 100% of its proceeds into more companies to support the goal of finding a cure for SCI. It is backed by five leading foundations in the United States, United Kingdom, and the European Union (EU [the Christopher & Dana Reeve Foundation, the Wings for Life, the International Spinal Research Trust, the Promobilia Foundation, and the Shepherd Center]), which for the first time have come together behind one single investment vehicle. The fund is modeled after successful VPFs for other indications (e.g., Cure Duchenne; the T1D Fund; Cystic Fibrosis VPF). SCI Ventures is led by tech entrepreneur and investor, Adrien Cohen, who co-founded two “unicorn” companies: Tractable and Lazada. Adrien Cohen holds business degrees from international academic institutions, started his finance career with Goldman Sachs, is an active angel investor, and has turned his attention to SCI after his family was impacted by a SCI.

**Summary:** SCI Ventures represents a transformative approach to funding innovations in SCI treatment, harnessing the power of venture philanthropy to bridge the gap between academic research and clinical application. The fund operates on an evergreen basis, reinvesting profits to ensure sustained investment in SCI start-ups. Adrien Cohen, a seasoned tech entrepreneur with personal ties to SCI, leads the fund, leveraging his expertise in finance and start-up ecosystems to drive the fund’s mission. Cohen highlighted the challenges of attracting the resources of life science VC to SCI due to a lack of understanding and some misconceptions of the condition. He suggested that venture philanthropy could pique VC interest by reducing investment risks through the combined network and expertise of its backing foundations. SCI Ventures is meticulous in its investment strategy. It looks for companies with the potential for high impact in SCI treatment, favoring early-stage ventures to nudge them to the clinic. Cohen emphasized the importance of VC in sparking and accelerating innovation. He pointed out that the VC industry often lacks understanding of specific conditions, and requires an expert vehicle to educate them and de-risk the market. SCI Ventures aims to demonstrate the viability and potential for returns of investing in SCI, encouraging VCs to diversify their portfolios. The role of SCI Ventures is to bring expertise, network, and funding to the table, effectively lowering the barriers for VCs to invest in SCI. Cohen aspires to emulate the success of venture philanthropy in other medical fields, such as cystic fibrosis and type 1 diabetes, where such funds have led to substantial market activation and the development of new treatment options. Cohen’s personal journey into the SCI space began when a family member suffered an injury leading to paralysis. His ensuing interactions with the scientific community revealed the stark lack of treatment options and the critical need for increased funding. Cohen was introduced to the Reeve Foundation, which had pioneered the venture philanthropy model with its investment in Onward Medical. This eventually led to the creation of SCI Ventures. The fund has a global remit. It targets technologies that can restore or replace lost functions and, in the long term, contribute to spinal cord repair. The fund’s criteria for investment encompass the maturity of the science, market potential, and the company’s technology potential for broader applications beyond SCI. Cohen stressed the need for SCI ventures to articulate the relevance of their work to other neurological conditions, expanding the market potential and appeal to VCs. He highlighted a case where a large German VC, traditionally uninterested in SCI, began considering SCI as a natural starting point for regenerative therapies that could later be applied to broader neurological conditions.

**Key points:** In summary, SCI Ventures, under Cohen’s guidance, is pioneering a strategic funding model to catalyze innovation in SCI. By bringing expertise and network, the fund seeks to mitigate investment risks, attract VC attention, and ultimately, contribute to the development of a cure for spinal cord injuries. Cohen’s conviction is that SCI Ventures can significantly influence the trajectory of SCI treatment development and encourage a new wave of entrepreneurs to bring their laboratory innovations to the real world.

## Conclusions

The discussions among leading SCI researchers at the workshop provided valuable insights into the complex process of translating basic science into clinical applications. While the stories shared by the researchers are undoubtedly interesting, it is crucial to critically analyze what worked, what did not, and why. The key lessons learned are the following.

### Identifying molecular targets early

#### What worked

Early identification of suitable molecular targets and developing a clear TPP were essential steps for successful translation, as seen in Dr. Strittmatter’s and Dr. Schwab’s journeys.

#### Challenges

Translating these targets into viable clinical treatments is fraught with difficulties, including the need for extensive preclinical studies, overcoming regulatory hurdles and accessing appropriate resources.

### Engaging with regulatory agencies

#### What worked

Early and continuous engagement with regulatory bodies like the FDA helped streamline the approval process. Dr. Anderson’s approach of delaying company formation afforded the efficient establishment of a fulsome preclinical profile without competing corporate aims.

#### Challenges

Regulatory requirements prioritize safety over efficacy, and navigating these demands requires significant expertise and resources.

### Collaboration and interdisciplinary efforts

#### What worked

Collaboration with experienced teams, consultants, and business development experts were crucial. Dr. Ruban’s methodical approach and use of multiple animal models to inform clinical trial design highlights the importance of interdisciplinary efforts.

#### Challenges

The lack of standardized preclinical testing infrastructure and the need to educate CROs on specialized techniques remain significant barriers.

### Funding and financial strategies

#### What worked

Utilizing diverse funding sources, including VC, grants, and philanthropic organizations, was vital for progressing through the translational pipeline. The support from organizations such as the Wings for Life ATP and SCI Ventures provided critical financial backing where resources were lacking.

#### Challenges

The high cost of translation and the need for innovative funding mechanisms to reduce timelines and attract investor interest are ongoing issues.

Despite the very different completed or partial journeys of five independent translational scientists, the lessons learned fall into some common themes. To expedite the process, it is important to identify suitable molecular targets and therapeutic agents as early as possible, meaning an early development of the TPP. Engaging with regulatory agencies early and understanding their requirements, which often focus primarily on safety and in Phase 2 and 3 on robust, clinically meaningful functional and quality of life improvements, is crucial. Collaborating with experienced teams, consultants, and business development experts is essential to cover areas of expertise that are usually not available in an academic environment. Protecting intellectual property is key to facilitating commercialization. Seeking funding from various sources, including VC, grants, and philanthropic organizations, is also important. In that regard, novel funding mechanisms such as the Wings for Life ATP and SCI Ventures can expedite translational research and clinical trials (see Table 2). Funding availability will be the biggest driver in the decision on when to create a company and start exploiting the therapeutic agents (see Table 3). In the rare case in which funding is potentially readily available (e.g., CIRM in California), delaying the creation will preserve the integrity and targeted direction of the therapy’s development. Balancing academic pursuits with entrepreneurial ambitions will be a challenge, and will always come at a cost, considering that publishing and conference participation will be hindered by the need to protect intellectual property. One should also consider the complexities introduced when forming a company, such as conflicts of interest and investor expectations. Managing both roles is nearly impossible, and it requires the formation of dedicated management teams and personal reflection on goals and the specific circumstances of each project.

**Table 2.** Supports for Translation

Bench to bedside translation	Primary activities	Traditional supports	Evolving supports
Discovery science ↓	Basic research, which may not have “practical goals,” results in general knowledge that provides partial answers to specific questions.	Academic, government, nongovernmental (e.g., SCI Foundations), and private philanthropy.	Private funding sources have increased as US Federal Government funding for R&D has declined by 13% since 2011.
Hit discovery and hit-to-lead optimization ↓	Chemical, molecular, or formula optimization. This is typically the step where much of the IP is created.		
Validation of animal models ↓	Applied research, which utilizes multiple models, provides answers to specific questions.		
*This is the typical timeframe to obtain IP and spin-off as biotech, to attract the funding sources.*			
Preclinical drug studies ↓	Solubility, stability, analytical methods/assays, *in vitro* mechanism of action, dose-response curves, pharmacokinetics (*in vivo*, C_max_, T_max_, T_½_, AUC), and PD.	• Government (e.g., NIH, NHS, EU Framework Programme for Research and Innovation). • Nongovernmental, including SCI Foundations. • Biotech companies funded by private philanthropy, venture capitalists, angel investors. • IP acquired by pharmaceutical companies.	2015: With big pharma shifting away from spinal cord injury, WFL identified a need for larger funding support of SCI programs that mapped out all the necessary steps to achieve a clinical trial (Accelerated Translational Program; ATP), e.g., ReNetX, NISCI, NervGen. 2024: With big pharma and venture capitalists still not considering SCI profitable, the SCI venture fund (SCI VF) was formed to assist in expediting therapeutic development specifically for SCI.
Clinically relevant technologies ↓	GMP production of drug products and GLP toxicology as regulatory requirements in preparation for clinical trials.		
Phase 1 and 2 clinical trials ↓	Human proof of concept, dose-response, safety, and preliminary efficacy.		
Bedside to practice translation:			
Phase 3 clinical trials	Establishes efficacy in humans and can influence the development of clinical guidelines.	IP acquired by Pharma (e.g., Acorda Therapeutics, Fampridine-SR, pivotal trials for SCI spasticity were unsuccessful, while approval was obtained for walking speed and lower extremity strength in MS).	2025: WFL ATP and SCI VF will expand as necessary to continue accelerating promising therapeutics into practice.

NIH, NHS, EU, European Union; AUC, PD, pharmacodynamics; WDL, WFL, SCI, spinal cord injury; GMP, good manufacturing practice; GLP, good manufacturing practice; IP, ATP, NISCI, Nogo-A Inhibition in acute Spinal Cord Injury. NIH (National Institutes of Health), NHS (National Health Service), EU (European Union), AUC (area under the curve), PD (pharmacodynamics), WFL (Wings for Life), SCI (spinal cord injury), GMP (good manufacturing practice), GLP (good manufacturing practice), IP (Intellectual Property), ATP (Accelerated Translation Program), NISCI (Nogo-A Inhibition in acute Spinal Cord Injury).

**Table 3.** How to Start a Spin-off Biotech Company for SCI

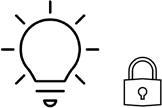 1. IP/Tech Transfer	Assess or obtain your IP status and license agreement with your organization’s tech transfer office. Write a business plan.
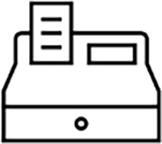 2. Estimate expenses	Evaluate your expenses for the next decade or more: facilities (incubator or your own), staff salaries, equipment and software (e.g., to manage the regulatory process).
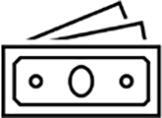 3. Source funding	Assess your funding sources: angel investors, venture capitalists, academic partners, nongovernmental agencies, and foundations, as well as other pharmaceutical companies.
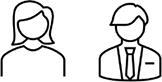 4. Obtain professional assistance	Choose a regulatory consultant to assist with financial decisions, data review and gap analysis, facilitation of research and commercial relationships, and sourcing suppliers for GMP/GLP and packaging.
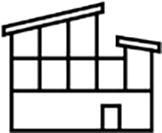 5. Engage regulatory agency	Request a PreIND meeting with your federal regulatory agency (e.g., see ^Table 3^ for essential elements of a PreIND meeting request letter).
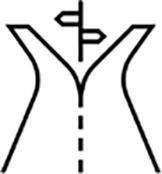 6. Create a road map	Based on your PreIND meeting, create a roadmap from preclinical activities to postapproval marketing.

SCI, spinal cord injury; GMP, good manufacturing practice; GLP, good manufacturing practice.

The SCI field faces unique challenges such as fitting into generic drug development models and educating contract research organizations (CROs) on specialized experiments. To provide assistance, a collaborative approach involving foundations and grant funding organizations can support the training of CROs and establish a standardized preclinical testing infrastructure, thus supporting the ecosystem and allowing more efficient testing for new candidates. Translational science requires purpose driven preclinical studies guided by a plan to add knowledge to an identified need in a particular disease state. The problem of “attrition” (i.e., failure of most drugs for human testing) has been debated for at least two decades and centers around the disparate physiology and anatomy between research animals and humans (Garner JP 2014; Verstappen K 2022).^3,12^ As a result, regulators have focused more on animal safety data (and less on animal efficacy data) in determining the fate of a drug candidate for human testing.

Evidence seems to indicate that the clinical SCI field is at the same stage that Multiple Sclerosis was in the seventies (Wynn DR 2019).^13^ There is no approved drug therapy to aid recovery, and therefore pharmaceutical companies are not interested. There is no demonstrated market path. This is one area where the ATP and SCI Ventures could greatly accelerate the translational process. If one drug achieves success, it will be much easier to push forward so-called “me-too” pharmaceutical products. Those might be academically less interesting but are attractive to pharma companies, which is an important goal if we want to accelerate the forward momentum in the SCI field.

## Transparency, Rigor, and Reproducibility Statement

The information summarized in this article adheres to the highest standards of transparency and rigor. All authors conducted thorough reviews and validation to maintain scientific integrity and reliability.

## Abbreviations Used

ATP: Accelerated Translation Program
AUC: Area Under the Curve
AXER-204: Soluble Nogo-Receptor-Fc decoy
BNN: Blueprint Neurotherapeutics Network
CIRM: California Institute for Regenerative Medicine
CMC: Chemistry, Manufacturing, and Control
CNS: Central Nervous System
CROs: Contract Research Organizations
EIC: European Innovation Council
EU: European Union
FDA: Food and Drug Administration
GLP: Good Laboratory Practice
GMP: Good Manufacturing Practice
hCNS-SC: human Central Nervous System Stem Cells
IIT: Investigator-Initiated Trial
IMPD: Investigational Medicinal Product Dossier
IND: Investigational New Drug Application
IP: Intellectual Property
IRBs: Institutional Review Boards
IT: intrathecal
LP: lumbar puncture
NCATS: National Center for Advancing Translational Sciences
NHS: National Health Service
NIH: National Institutes of Health
NINDS: National Institute of Neurological Disorders and Stroke
NISCI: Nogo-A Inhibition in acute Spinal Cord Injury
PD: Pharmacodynamics
PK: Pharmacokinetics
RESET: Renetx Safety Efficacy and Tolerability of Axer-204 for chronic SCI
SCI: Spinal Cord Injury
SOPs: Standard Operating Procedures
TPP: Target Product Profile
TRL: Technology Readiness Level
TTO: Technology Transfer Office
VC: Venture Capital
VPF: Venture Philanthropy Fund
WFL: Wings for Life
